# Validation of Tampa Scale for Kinesiophobia in patients with shoulder instability

**DOI:** 10.1002/jeo2.70349

**Published:** 2025-07-13

**Authors:** Marcello Motta, Alessandra Scaini, Maristella Francesca Saccomanno, Andrea Bergomi, Almerico Megaro, Valerio Daffara, Giuseppe Milano

**Affiliations:** ^1^ Department of Medical and Surgical Specialties, Radiological Sciences, and Public Health University of Brescia Brescia Italy; ^2^ Department of Bone and Joint Surgery ASST Spedali Civili Brescia Italy

**Keywords:** kinesiophobia, questionnaire, reliability, shoulder instability, Tampa scale, validity

## Abstract

**Purpose:**

The Tampa Scale for Kinesiophobia (TSK) has been used to evaluate psychological outcomes in patients with low back pain, anterior cruciate ligament injuries, and other orthopaedic conditions, but no previous study verified the validity of TSK for patients with glenohumeral instability. The purpose of this study was to evaluate the measurement properties of the Tampa Scale of Kinesiophobia (TSK‐13) in patients with glenohumeral instability.

**Methods:**

The present study was designed as an observational study that includes individuals with a diagnosis of pathological glenohumeral instability. All patients underwent a structured interview to collect information on personal socio‐demographic and contextual characteristics at the time of enrolment. Kinesiophobia was assessed using the 13‐item version of the TSK‐13. The questionnaire is divided into two domains (subscales): activity avoidance (AA) and health anxiety (HA). Additionally, enroled patients were administered the American Shoulder and Elbow Society (ASES) score and the Western Ontario Shoulder Instability Index (WOSI) questionnaire. The validation of the TSK questionnaire was conducted according to the analysis plan outlined in the IQOLA project and current guidelines. Correlation analysis was performed between TSK and ASES and WOSI.

**Results:**

The study population consisted of 100 patients. The TSK‐13 questionnaire showed no floor and ceiling effects; all the correlations between each question in a given domain and the score of the same domain showed a very significant correlation (*p* < 0.0001) with Pearson's correlation coefficient greater than 0.90. Regarding discriminant validity, for each domain, 100% of questions showed a higher correlation with the domain of belonging than with the other domains. Correlation analysis between the TSK and the other questionnaires showed a significant correlation between each domain (and the overall score of TSK‐13 and ASES and WOSI questionnaires. The internal consistency was good for each domain and for the overall score (Cronbach's *α* = 0.874, 0.787, and 0.851 for AA and HA and the overall score, respectively). Test‐retest reliability was excellent for both domains (ICCs = 0.927 and 0.878 for AA and HA domains, respectively), and the overall score (ICC = 0.915).

**Conclusion:**

Measurement properties of the TSK‐13 in patients with glenohumeral instability were good to excellent in terms of validity and reliability. The TSK‐13 is a valid and useful instrument to assess kinesiophobia in patients affected by glenohumeral instability.

**Level of Evidence:**

Level III, observational study.

**Abbreviations**
AA: activity avoidance
ACL: anterior cruciate ligament
ASES: American Shoulder and Elbow Society score
HA: health anxiety
ICC: intraclass correlation coefficient
PROMs: Patient Reported Outcome Measures
QoL: quality of life
RTS: return to sport
TSK‐13: Tampa Scale of Kinesiophobia 13
WOSI: Western Ontario Shoulder Instability Index questionnaire

## INTRODUCTION

Kori et al. [29] have defined kinesiophobia as 'an irrational and debilitating fear of physical activity and movement resulting from a sense of vulnerability to painful injuries or setbacks'. In literature, the terms 'ear of movement' and 'kinesiophobia' are often used interchangeably.

Patients with kinesiophobia avoid performing certain movements and activities of daily life out of 'fear'. When this happens, the patient can experience increased pain and discomfort while engaging in specific physical activities [9, 43, 48].

The role of kinesiophobia has already been investigated in association with various musculoskeletal conditions, and it has been highlighted that patients with high levels of fear of movement achieve significantly worse outcomes in terms of functionality and residual pain in various post‐surgical contexts, such as knee joint replacement surgery and lumbar disc herniation surgery [3, 20]. In particular, kinesiophobia appears to play a fundamental role in returning to sports activities after anterior cruciate ligament reconstruction surgery [39].

Regarding shoulder surgery, an association between kinesiophobia and functional and subjective outcomes in the treatment of rotator cuff injuries has been reported [26]. A recent systematic literature review has shown that high levels of kinesiophobia predict greater pain and disability over time in patients with various shoulder pathologies [32]. Among these, glenohumeral instability is an extremely common condition in young athletes [15, 16], characterised by recurrent episodes of shoulder dislocation and subluxation.

The failure of conservative or surgical treatment for glenohumeral instability is not only assessed in terms of dislocation recurrence (objective outcome), but also concerning the patient's subjective perception of joint instability, which generates a strong sense of apprehension, and thus 'fear', that certain movements of the limb may cause a new episode of dislocation or subluxation [32].

One of the most commonly used tools for measuring kinesiophobia is the Tampa Scale of Kinesiophobia (TSK) [36]. Although the TSK has been employed to assess various orthopaedic conditions and treatments [5, 7, 31, 39, 51], it has not yet been validated in a population of individuals with glenohumeral instability.

The purpose of the current study was to validate the TSK‐13 in a population of individuals with glenohumeral instability. The study hypothesis is that the TSK is valid for assessing kinesiophobia in patients with glenohumeral instability.

## METHODS

### Study design

The present research was designed as an observational study. The local IRB and Ethic Committee approved the study protocol. The study was conducted according to the principles of good clinical practice and the Declaration of Helsinki and its updated version (Tokyo 2004).

### Study population

Patients were selected from the electronic databases of the Emergency Department and outpatient clinics at the Orthopaedics and Traumatology Unit of ASST Spedali Civili in Brescia, Italy. The inclusion criteria were: a clinical diagnosis of pathological glenohumeral instability, regardless of the cause (traumatic or atraumatic), direction (anterior, posterior or multidirectional), and frequency (acute or recurrent); and age over 18 years (with no upper age limit). Patients who consented to participate and met the inclusion criteria were enroled. The following exclusion criteria were considered: previous fractures or surgeries on the affected shoulder; previous or ongoing tumour or infections affecting the shoulder; systemic or local neurological or inflammatory disorders; inability to complete the questionnaires due to linguistic and/or cognitive issues.

All patients underwent a structured interview to collect information on personal socio‐demographic and contextual characteristics at the time of enrolment. Additionally, all patients were asked to complete self‐administered questionnaires, with instructions provided by one of the researchers involved in the study.

### Questionnaire administration

Enroled patients were administered the American Shoulder and Elbow Society (ASES), and the Western Ontario Shoulder Instability Index (WOSI) questionnaires.

The ASES questionnaire [41] consists of a self‐assessment section that includes visual analogue scales (VAS) for pain and instability, along with a questionnaire about activities of daily living. The latter is based on a 4‐point scale that can be converted into a cumulative index of daily life activities. The overall score can be derived from the VAS score for pain (50%) and the cumulative score of daily life activities (50%).

The WOSI questionnaire [27] (was designed to assess the quality of life (QoL) in patients with shoulder instability. It consists of 21 questions, divided into five domains: symptoms (six questions), sports/recreational activities (four questions), work (four questions), lifestyle (four questions) and emotional aspects (three questions). The score for each question is evaluated on a Visual Analogue Scale (VAS) ranging from 0 (minimal disability, best result) to 100 points (maximum disability, worst result). The overall score, therefore, ranges from 0 to 2100 points, where a higher score indicates a worse QoL. Through arithmetic conversion, the overall score is expressed as a percentage.

Furthermore, all patients were administered the Tampa Scale of Kinesiophobia (TSK‐13) questionnaire [38] which consists of 13 items evaluated using a 4‐point Likert scale and is divided into two subscales: Activity Avoidance (TSK‐A), which reflects the belief that activity can lead to setbacks or increased pain, and Harm (TSK‐H), which reflects the belief of having severe health problems. The total scale result consists of a total score and two subscale scores. The maximum total score is 52 points, while the subscale scores are 24 points for TSK‐A and 28 points for TSK‐H. A higher score indicates a higher degree of kinesiophobia.

The ASES and WOSI questionnaires were administered once, while the TSK‐13 questionnaire was administered again to each patient 2 weeks after the initial administration (re‐test). All questionnaires were administered in their respective validated national versions [6, 37, 40].

### Outcome measures

The validation of the TSK questionnaire was conducted according to the analysis plan outlined in the IQOLA project and current guidelines [4, 17, 44, 50]. This phase aimed to verify the questionnaire's validity in the specific context of the study using the following outcome measures.

#### Content validity

Verification of the hypotheses underlying the construction of the questionnaire items through a series of psychometric tests assessing the adequacy of the questions and the correlation between the questions and their respective domains.

#### Construct validity

Verification of the correlation between the TSK questionnaire and other questionnaires assessing similar hypotheses.

#### Reliability

Verification of the questionnaire's reproducibility.

### Statistical analysis

All data were analysed using SPSS software version 26 (IBM Statistics, Harmonk, NY, USA). The sample size was determined based on current guidelines [4, 44], by enroling a minimum sample of 100 patients.

Descriptive statistics were reported for each item (question) and domain (subscale). For items, the percentage and distribution of missing data were reported; the mean and standard deviation (SD) of each question and the distribution of responses for each question in each domain were also reported. For domains, the mean and standard deviation of each domain, the ratio of observed minimum values to possible minimum values, and the ratio of observed maximum values to possible maximum values were reported to determine ceiling and floor effects. Those were confirmed if more than 15% of patients reached the lowest or highest possible score, respectively [34, 44].

Content validity was assessed using multi‐trait analysis, a psychometric technique examining the correlation between each question and its hypothetical domain, as well as the correlation between each question and other domains. Internal consistency of questions, equality of item‐scale correlation, and item discriminant validity were tested.

Construct validity was tested by analysing the correlation between the TSK questionnaire and the ASES and WOSI questionnaires [14].

Reproducibility was evaluated using two methods: internal consistency and test‐retest reliability. Internal consistency was assessed by calculating Cronbach's alpha coefficient for each domain, with values > 0.70 indicating moderate reproducibility [46]. Test‐retest reliability was evaluated using the intraclass correlation coefficient (ICC), with values between 0.50 and 0.75 indicating moderate reliability and values between 0.75 and 0.90 indicating good reliability, while values > 0.90 indicating excellent reliability [28].

## RESULTS

### Study population

The study population consisted of 100 patients. Demographic data of the study population is reported in Table 1.

**Table 1:** Demographic data of study population.

Variables		*N* = 100
Age (years)	Mean ± SD	27.9 ± 9.4
Gender	Male, *N* (%)	83 (83%)
	Female, *N* (%)	17 (17%)
Dominance	No, *N* (%)	43 (43%)
	Yes, *N* (%)	57 (57%)
N. of dislocations	Mean ± SD	7 ± 6
Timing (months)	Mean ± SD	28.5 ± 22.6
Hyperlaxity	No, *N* (%)	74 (74%)
	Yes, *N* (%)	26 (26%)
Type of work	Manual, *N* (%)	16 (16%)
	Sedentary, *N* (%)	84 (84%)
Type of sport	No, *N* (%)	10 (10%)
	Contact, *N* (%)	41 (41%)
	Non contact, *N* (%)	37 (37%)
	Overhead, *N* (%)	12 (12%)
WOSI score	Mean ± SD	36.1 ± 18.8
ASES score	Mean ± SD	60.7 ± 13.6
TSK‐13	Mean ± SD	23.6 ± 8.9

Abbreviations: ASES, American Shoulder and Elbow Society score; SD, standard deviation; TSK‐13, Tampa Scale of Kinesiophobia 13; WOSI, Western Ontario Shoulder Instability Index questionnaire.

### Descriptive statistics (item level)

The descriptive statistics data are shown in Table 2. The absence of missing data confirms good understanding of the questionnaire by the patients. The Shapiro–Wilk test confirmed normal distribution of data for each item. Being a young active population of patients suffering from shoulder disorder but in good health status, the distribution of responses in most items was directed towards higher (better) scores.

**Table 2:** Item descriptive statistics.

Items	Missing (%)	Mean	SD	Response values frequency			
				1	2	3	4
Subscale = Activity avoidance (TSK‐A)							
Q‐1	0	2.1	1	38	29	23	10
Q‐2	0	1.6	09	59	27	8	6
Q‐8	0	1.8	1	56	24	9	11
Q‐11	0	1.4	0.8	79	8	9	4
Q‐12	0	1.6	0.9	61	23	10	6
Q‐13	0	2.1	1	32	37	18	13
Subscale = Harm (TSK‐H)							
Q‐3	0	1.7	1	62	17	14	7
Q‐4	0	1.5	0.9	71	14	10	5
Q‐5	0	2	1.1	50	16	22	12
Q‐6	0	1.9	1	46	28	14	12
Q‐7	0	2.3	1.1	36	20	26	18
Q‐9	0	1.5	0.9	74	12	9	5
Q‐10	0	2.4	1.1	31	20	31	18

Abbreviations: SD, standard deviation; TSK‐A, Tampa Scale of Kinesiophobia‐Activity Avoidance; TSK‐H, Tampa Scale of Kinesiophobia‐Harm.

### Descriptive statistics (scale level)

For each domain, mean and standard deviation, minimum and maximum values and range, and proportion of lowest (floor) and highest (ceiling) responses were calculated (Table 2). The answers used the possible scores extensively; no floor or ceiling effects were found for the two subscales.

### Item internal consistency and equality of item‐scale correlation

All the correlations between each question in a given domain and the score of the same domain (corrected for overlap) showed Pearson's coefficient greater than 0.90 showing a significant correlation (*p* < 0.0001). All the questions in each domain contribute equivalently to the total score of the domain (Table 3).

**Table 3:** a–b: Item internal consistency.

a)		
Item	Scales	
	ACT	HARM
Subscale = Activity avoidance (TSK‐A)		
Q‐1	0.982*	0.790*
Q‐2	0.983*	0.790*
Q‐8	0.983*	0.776*
Q‐11	0.989*	0.801*
Q‐12	0.990*	0.786*
Q‐13	0.972**	0.814*
Subscale = Harm (TSK‐H)		
Q‐3	0.800*	0.991*
Q‐4	0.782*	0.990*
Q‐5	0.820*	0.990*
Q‐6	0.797*	0.988*
Q‐7	0.800*	0.986*
Q‐9	0.794*	0.993*
Q‐10	0.794*	0.983*

b)		
Item	Scales	
	ACT	HARM
Subscale = Activity avoidance (TSK‐A)		
Q‐1	[**]	1
Q‐2	[**]	1
Q‐8	[**]	2
Q‐11	[**]	1
Q‐12	[**]	2
Q‐13	[**]	1
Subscale = Harm (TSK‐H)		
Q‐3	1	[**]
Q‐4	2	[**]
Q‐5	1	[**]
Q‐6	1	[**]
Q‐7	1	[**]
Q‐9	1	[**]
Q‐10	1	[**]

*Note*: Item‐scale correlations corrected for overlap (relevant item removed from its scale for correlation); item discriminant validity test. Cutoff point for significance is 2 standard errors.

Levels of scaling success: 2: Item‐scale correlation is significantly higher for hypothesised scale than for competing scale. 1: Item‐scale correlation is higher for hypothesised scale than competing scale, but not significantly. −1: Item‐scale correlation is lower for hypothesised scale than competing scale, but not significantly. −2: Item‐scale correlation is significantly lower for hypothesised scale than for competing scale.

Abbreviations: TSK‐A, Tampa Scale of Kinesiophobia‐Activity Avoidance; TSK‐H, Tampa Scale of Kinesiophobia‐Harm.

* Statistically significant correlation (*p* < 0.01).

** Discriminant validity test not conducted.

### Item discriminant validity

The correlation between the question and the domain to which it belonged was significantly higher than the correlation between the same question and the other domains in all cases (Table 3). The percentages of questions that showed a higher correlation with the domain of belonging than with the other domains was 100% for each domain (Table 4).

**Table 4:** Frequency and percentage of Item‐scale correlations at each level of scaling success.

Subscales	−2		−1		1		2		1 + 2	
	*n*	%	*n*	%	*n*	%	*n*	%	*n*	%
Activity avoidance (TSK‐A)	0	0	0	0	4	66.7	2	33.3	6	100
Harm (TSK‐H)	0	0	0	0	6	85.7	1	14.3	7	100

*Note*: Levels of scaling success: 2: Item‐scale correlation is significantly higher for hypothesised scale than for competing scale. 1: Item‐scale correlation is higher for hypothesised scale than competing scale, but not significantly. −1: Item‐scale correlation is lower for hypothesised scale than competing scale, but not significantly. −2: Item‐scale correlation is significantly lower for hypothesised scale than for competing scale.

Abbreviations: TSK‐A, Tampa Scale of Kinesiophobia‐Activity Avoidance; TSK‐H, Tampa Scale of Kinesiophobia‐Harm.

### Construct validity

Correlation analysis between the TSK and ASES and WOSI questionnaires showed a significant correlation between each domain of TSK and the overall score, and ASES and WOSI questionnaires. The correlation between TSK‐A and ASES showed a Pearson's coefficient (*r*) of 0.661 with a *p* < 0.0001 while for the WOSI the Pearson's coefficient (*r*) was −0.501 with a *p* < 0.0001; the correlation between TSK‐H and ASES showed a Pearson's coefficient (*r*) of 0.683 with a *p* < 0.0001 while for the WOSI the Pearson's coefficient (*r*) was −0.501 with a *p* < 0.0001. The overall correlation between TSK and ASES showed a Pearson's coefficient (*r*) of 0.707 with a *p* < 0.0001 while for the WOSI the Pearson's coefficient (*r*) was −0.532 with a *p* < 0.0001.

### Reproducibility

Internal consistency was calculated using Cronbach's alpha, with data obtained from the administration of the questionnaire to all patients. The internal consistency was > 0.80 for the TSK‐A domain and for the overall score and > 0.70 for the TSK‐H domain (Table 5).

**Table 5:** Reliability coefficients and inter‐scale correlations at test‐retest evaluation.

Scales	TSK‐A	TSK‐H	TSK
Subscale = Activity avoidance (TSK‐A)	0.874*	0.672*	0.809*
Subscale = Harm (TSK‐H)	0.735*	0.787*	0.814*
TSK overall (TSK)	0.836*	0.772*	0.851*

*Note*: Scale internal consistency reliability (Cronbach's alpha coefficient) is presented in the diagonal.

Abbreviations: TSK‐A, Tampa Scale of Kinesiophobia‐Activity Avoidance; TSK‐H, Tampa Scale of Kinesiophobia‐Harm.

* Statistically significant correlation (*p* < 0.01).

The ICC values were greater than 0.80 for both subscales; the subscale TSK‐A showed an ICC of 0.927 (0.850–0.964) while the subscale TSK‐H showed an ICC of 0.878 (0.750–0.941). The overall reliability of the TSK was good as well, with a value of 0.915 (0.825–0.958).

## DISCUSSION

The main findings of the present study were that TSK‐13 is a valid and reliable tool to assess kinesiophobia in patients affected by glenohumeral instability.

The validation process of TSK is clinically relevant in order to provide a tool to assess and quantify the severity of fear of movement in patients suffering from glenohumeral instability. Identification of patients with kinesiophobia is the first step providing a solution to reduce fear of movement, which may eventually lead to improved patient's satisfaction, patient‐reported outcome and health‐related QoL.

The TSK was adapted for evaluation of kinesiophobia in several musculoskeletal conditions such as ACL injuries [22, 30], total knee replacement [5] and temporomandibular disorders [18]. Nevertheless, TSK has been used to evaluate the outcome of shoulder surgery, such as rotator cuff repair [49] and shoulder stabilisation surgery; [47] however, the questionnaire has not been validated in populations of individuals with shoulder instability, and therefore data explanation from those studies [29] needs careful interpretation.

The assessment of kinesiophobia in patients affected by glenohumeral instability is very important; in fact glenohumeral instability can result in sport time‐loss (e.g., missed games) and high socioeconomical and hospital costs [11].

Even though return to sport (RTS) rates in literature are quite good, up to 19% of patients with anterior shoulder instability are unable to RTS [1, 23, 24, 35, 45], despite advances in rehabilitation and surgical technique and it's mandatory for clinicians and patients also to understand the reasons behind this.

The ability of the patient with shoulder instability to RTS is a multifactorial process. In the last few years, the relation between fear for shoulder movements (kinesiophobia) and return to preinjury level of sport has been progressively investigated, highlighting that the reason not to RTS could be psychologically motivated and not only be shoulder function dependent [47].

In light of this, a recent study showed that 74% of these patients failed to RTS because of reasons independent of the shoulder, such as fear for reinjury, kinesiophobia, and concerns about a new rehabilitation process; [42] these results are also confirmed by a systematic review [19] where up to 85.1% of the patients, after surgery for shoulder instability, had a psychological reason for not returning to sport.

Fear of reinjury seems to be a key issue in sports injury rehabilitation and returning to the preinjury activity level and satisfaction [8, 25, 41].

Psychological assessment tools (e.g., TSK‐13) can assist clinicians (especially orthopaedic surgeons) in elucidating certain psychological factors that may affect an athlete's ability to return to sport, such psychological readiness, confidence and kinesiophobia; PROMs like ASES and WOSI assess QoL of patients, respectively with shoulder instability and shoulder diseases, but they don't investigate kinesiophobia, like TSK‐13, or other psychological features. Taking into consideration that in our study we found a significant correlation between each domain of TSK‐13 and questionnaires that assess QoL related to specific shoulder disabilities (ASES and WOSI), the assessment of Kinesiophobia levels, through TSK‐13, could explain some variable result in quality of life and RTP in patients affected by shoulder instability.

The TSK was originally developed on 17 items to assess kinesiophobia and the level of comfort, safety, and preparation for movement in patients with low back pain [36]. The abbreviated version TSK‐13 results from removing reverse items [7, 26, 33, 48] from the original scale as it appears to improve the psychometric characteristics of the outcome measure [2, 12, 38].

In the present study, TSK‐13 was confirmed to have good psychometric characteristics in the population of interest. The questionnaire was completed by all patients with no difficulties, and there were no items with multiple or missing answers indicating good cultural acceptability. A good distribution of responses was observed in the present study, which indicates no floor or ceiling effects.

Construct validity was assessed by determining the correlation between the TSK‐13 and ASES and WOSI scores. These questionnaires were selected because they are the most commonly used tools for self‐assessment of quality of life (QoL) related to specific shoulder disabilities. Furthermore, both questionnaires have demonstrated good internal consistency and reliability in tests conducted on patients with shoulder instability [13]. We found a significant correlation between each domain of TSK‐13 and questionnaires that assess QoL related to specific shoulder disabilities.

Reproducibility was assessed by administering the questionnaire twice, 14 days apart. This time interval was long enough to prevent recall bias but short enough to ensure that clinical changes had not occurred [44]. The internal consistency of the TSK‐13 was good with Cronbach's *α* of 0.874 for the total score, and slightly lower for the subscales (0.787 for TSK‐H and 0.851 for the TSK‐A). These scores are similar to those of other validation studies of TSK for varying pathologic conditions, such as sciatica due to disc herniation [21], low back pain [10, 37], and ACL injuries [22]. The test‐retest showed an excellent reproducibility, which concurred with previously published findings and suggested substantial reliability [37].

### Limitations

The main limitation of this study is related to its applicability only to patients with glenohumeral instability; in fact, it cannot be generalised to patients with other musculoskeletal conditions, including other shoulder diseases. Second, we did not examine the responsiveness of the TSK‐13; therefore, the extent to which the TSK‐13 can detect changes in kinesiophobia over time among patients with shoulder instability remains unclear. In addition, we did not analyse the clinical and imaging features of our population, to correlate the pattern and severity of injury of every patient with the levels of kinesiophobia.

## CONCLUSIONS

The TSK is a valid and reliable valid measurement tool to assess fear of movement in individuals with glenohumeral instability.

## AUTHOR CONTRIBUTIONS

Marcello Motta: Wrote the preliminary draft of the manuscript and contributed to data input. Alessandra Scaini: Wrote the study protocol. Maristella Fracesca Saccomanno: Supervised data gathering and input. Andrea Bergomi: Gathered primary data and contributed to data input. Almerico Megaro: Provided valuable assistance in the design of the project and the interpretation of the data. Valerio Daffara: Provided substantial assistance in the interpretation of the data and drafting of the manuscript. Giuseppe Milano: Generated the study hypothesis, performed data interpretation and revised the final version of the manuscript.

## CONFLICT OF INTEREST STATEMENT

The authors Marcello Motta, Maristella Francesca Saccomanno, Andrea Bergomi, Almerico Megaro and Valerio Daffara or any member of their immediate family, have no funding or commercial associations (e.g., consultancies, stock ownership, equity interest, patent/licensing arrangements, etc.) that might pose a conflict of interest in connection with the submitted article. Giuseppe Milano reports a relationship with Arthrex Inc. that includes consulting or advisory.

## ETHICS STATEMENT

The present study was designed as an observational study. The local Ethic Committee of the ASST Spedali Civili, Brescia, Italy approved the study protocol (NP 5221). The study was conducted according to the principles of good clinical practice and the Declaration of Helsinki and its updated version (Tokyo 2004). Informed consent was obtained from all individual participants included in the study.

## Data Availability

The data that support the findings of this study are available from the corresponding author upon reasonable request.
